# Multi-step thermal design of microwave vacuum heating to basaltic regolith simulant towards lunar base construction

**DOI:** 10.1038/s41598-024-79504-x

**Published:** 2024-11-15

**Authors:** Kunihiko Kato, Takashi Shirai

**Affiliations:** Advanced Ceramics Research Center, Nagoya Institute of Technology, Gokiso, Showa-ku, Nagoya, 466-8555 Aichi Japan

**Keywords:** Regolith, Microwave, Lunar base construction, Vacuum, FJS-1, Structural materials, Planetary science

## Abstract

Humanned exploration and extended stay on the Moon are the hottest challenges today. We report highly robust materials by microwave heating under high-vacuum conditions (10^− 3^ Pa) from a basaltic regolith simulant (FJS-1) to ensure the feasibility of lunar infrastructure construction. The violent degassing dynamics were revealed by monitoring vacuum pressure during heating and analyzing the compounds evolved from basaltic silicate compounds: thermal pyrolysis of silicate compounds became more pronounced above 1000 ^o^C, leading to a catastrophic deformation accompanied by forming countless pores of several hundred µm sizes. The preferential formation of the magnetite phase in vacuum heating dominantly caused the radical change in microwave absorption capacity compared with conventional heating routes using an electrical furnace in atmospheric conditions. Besides, the in-situ measurement of dielectric properties during microwave vacuum heating clarified the increase in one order of magnitude from 100 to 1000 ^o^C. Based on the presumed phenomena during microwave-vacuum heating for basaltic regolith, we propose a new thermal design concept to overcome the practical limitations of microwave technology. The multi-step temperature profile successfully fabricated a highly robust product that demonstrated world-class mechanical performance, equivalent to the compressive strength of 65 MPa without any vigorous hydrostatic cold press as a pre-treatment.

**Supplementary Information:**

The online version contains supplementary material available at 10.1038/s41598-024-79504-x.

## Introduction

Humanned exploration and extended stay on the Moon are the hottest challenges recently, and the ARTEMIS mission is just underway. Not to mention the extremely thin atmosphere (10^− 12^ − 10^− 15^ atm) and scarcity of water, several environmental factors make it challenging to explore and stay on the Moon, including the extreme temperature differences^1^ and the intense radiation from outer space^2^. Furthermore, even a tiny piece of sub-mm size produced by a secondary impact is comparable in power to a bullet that could easily penetrate a space suit in addition to the catastrophic effects of a milligram-scale meteoroid on hitting a sizeable lunar base^3^. Some foldable shelters have been commercially launched for short stays; however, it is necessary to construct a highly robust moon shelter or protective layer for the living quarters since the above risks will increase dramatically during an extended stay. As other troublesome issues, floating regolith particles covering the lunar surface retard the transportation efficiency of lunar rovers due to low gravity (1/6 G) with bumpy terrain^3^. In addition, the dust’s highly adhesive and abrasive nature can quickly wear down mechanical components such as motors and gears used in rovers, manipulators, drills, and manufacturing machinery, resulting in equipment failure. The construction of a lunar road will effectively improve the safety and efficiency of transporting people and resources, dramatically improving lunar scientific research activities. Thus, constructing lunar infrastructure is an essential and urgent task to improve safety, functionality, and operational efficiency.

A technology to manufacture construction materials using resources available on the Moon (In-Situ Resource Utilization; ISRU^4^) provides transportation opportunities for the highest priority items (e.g., water, food, life support equipment) from the Earth for realizing larger-scale humanned missions. The entire lunar surface is covered by a sedimentary layer of a few meters to a dozen meters (i.e., regolith). Meteorite impacts, solar winds, cosmic rays, and other cosmic weathering effects have created lunar regolith over hundreds of millions of years. Since the successful Apollo 11 lunar landing (July 1969), some soil has been returned to Earth and analyzed in detail. The regolith and soils are mainly composed of silicate minerals with a chemical composition similar to basalt, especially for mare regions^5,6^. Most of the methods of building materials fabrication using regolith are classified into three types (summarized in Table S1, in Supplementary Information (SI)): Solidification of regolith through chemical reactions such as concrete and geopolymer is one of the most notable routes with empirical knowledge in fabrication capability as terrestrial applications^7,8^. However, these methods have fatal disadvantages, requiring scarce resources such as water and substances not found on the Moon (i.e., organic binder), in addition to facilities to prevent water evaporation until complete curing. On the other hand, hardening methods such as cold press have a prospective feature for practical construction on Mars^9^, a requirement of clay minerals is unsuitable for use on the Moon. Sintering/melting methods using a microwave oven, electric furnace, and laser system can achieve the highest strength blocks without additives^10–12^. However, it should be noted that sophisticated know-how is required for temperature control in high-temperature regions. In addition, in the extremely high vacuum on the lunar surface, conventional electric furnaces using thermal convection and thermal conduction greatly suffer from the inefficiency of heating due to the low thermal conductivity of basaltic materials (7.4 × 10^− 4^ ~ 3.4 × 10^− 3^ W m^− 1^K^− 1^)^13^; about 1/70,000 to 1/320,000 of aluminum). Recently, microwaves, whose a frequency range of 300 MHz to 300 GHz, have attracted much attention as a prominent heat source to achieve efficient heating even in a vacuum environment due to self-heating by direct interaction between the microwave electromagnetic field and the material, as well as capability of penetrating into the regolith to a depth of 65 cm^10^. Although the conception of lunar infrastructure using microwave technologies has long been proposed^10^, Many studies have pointed out that the forming pores causing structural defects are inevitable in a vacuum environment^14,15^, because of the prominent gases generated by thermal pyrolysis in the high-temperature range^16^. A hybrid heating method using a microwave furnace and an electric furnace has been proposed recently^17^; however, the antinomic issue limits the feasibility of using the microwave process despite the incomparable potential to achieve high strength.

Here, we first report highly robust materials by 2.45 GHz microwave heating under high-vacuum conditions (10^− 3^~ Pa) only from a regolith simulant (FJS-1). The influence of fabrication conditions (e.g., treatment temperature and atmosphere) on the microstructure and chemical structure of the obtained specimens was investigated. It revealed the degassing dynamics that lead to fatal structural defect formation by monitoring vacuum pressure during heating and analyzing the volatile compounds evolved from basaltic silicate compounds. Moreover, the radical change in microwave absorption capacity was extensively evaluated from the following perspectives: the heat generation behavior derived from the interaction between the silicate compounds and microwave electric/magnetic components, a comparison in specimen’s dielectric properties obtained through various synthetic conditions, and in-situ measurement of microwave dielectric properties in a vacuum condition. Based on the investigation, we discussed the possible phenomena during microwave-vacuum heating for basaltic regolith. In the last section, we propose a new thermal design concept to overcome the practical limitations of microwave technology in a vacuum atmosphere. The multi-step temperature profile successfully fabricated a highly robust product that demonstrated world-class mechanical performance, equivalent to the compressive strength of 65 MPa despite no vigorous hydrostatic cold press as a pre-treatment.

## Results and discussion

### Microstructure

**Fig. 1:**
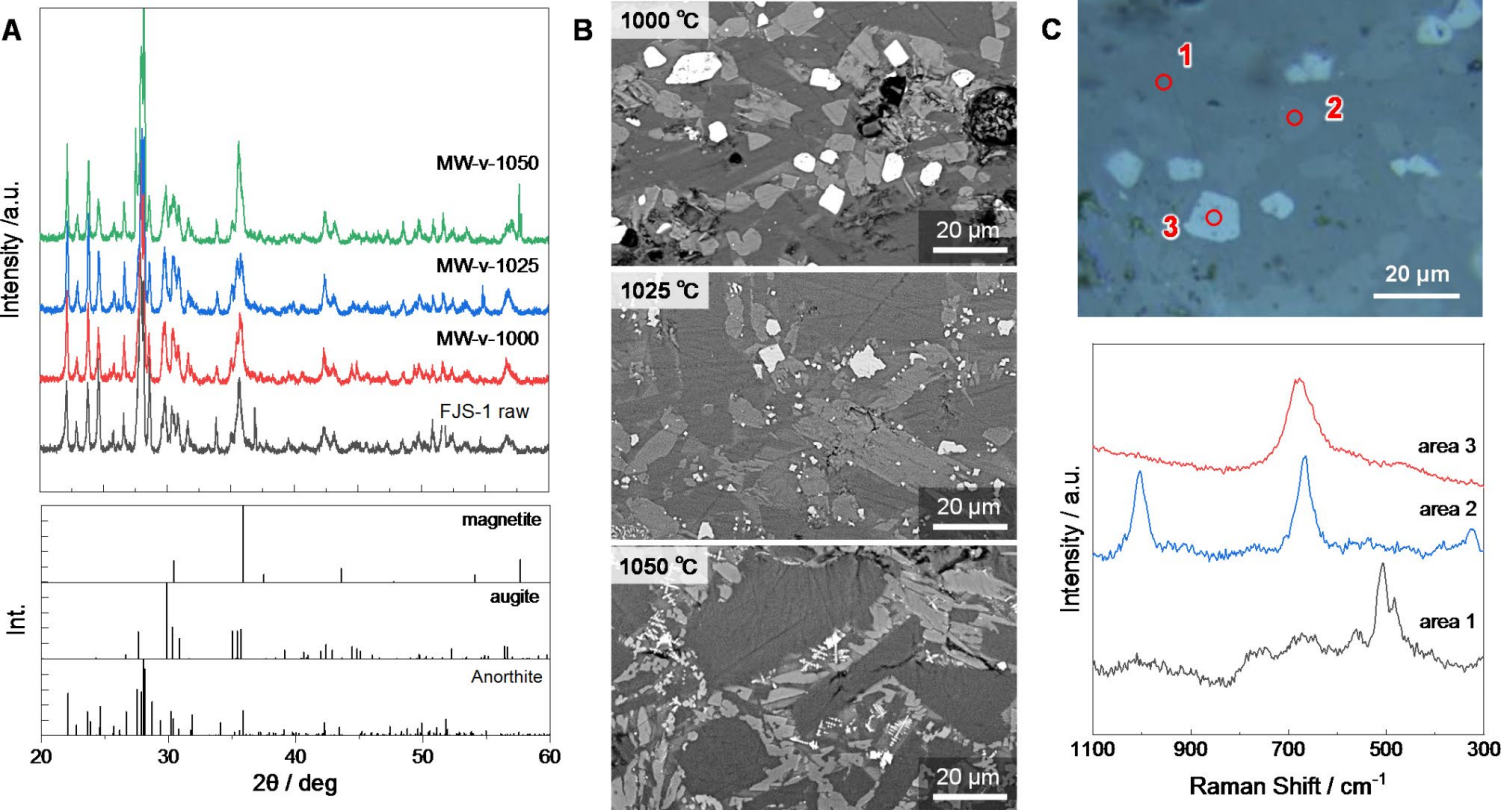
(**A**) PXRD patterns. (**B**) SEM-BSE images of polished cross-section of dense part. (**C**) Microscopic image and Raman spectra,

Figure 1A and Fig. S1 display the PXRD patterns of the obtained specimens through the vacuum and atmospheric conditions, respectively. The major components were identified as sodian anorthite ((Na, Ca)(Si, Al)_3_O_8_), pyroxene ((Ca, Mg, Fe)_2_Si_2_O_6_); pigeonite or augite), and magnetite (Fe_3_O_4_). The diffraction patterns of sodian anorthite shifted towards a lower angle after heating, associated with compositional changes to intermediate sodian anorthite^18^. Besides, the reducing atmosphere facilitated the crystallization of magnetite, whereas the heating in the oxidizing atmosphere resulted in the formation of the hematite (Fe_2_O_3_) phase. Figure 1B represents the microwave-heated specimen’s backscattered electron (BSE) images. The emission of BSE reflects a compositional difference in the specimen’s microstructure. The crystals containing heavier elements (i.e., Fe, Ti) show whiter contrasts, while darker contrast is observed in crystals composed only of lighter elements, such as Ca-Na, Al, and Si^19,20^. In this case, magnetite, pyroxene, and anorthite represented a brighter contrast in that order, identified by using Raman microscopy (Fig. 1C).

**Fig. 2:**
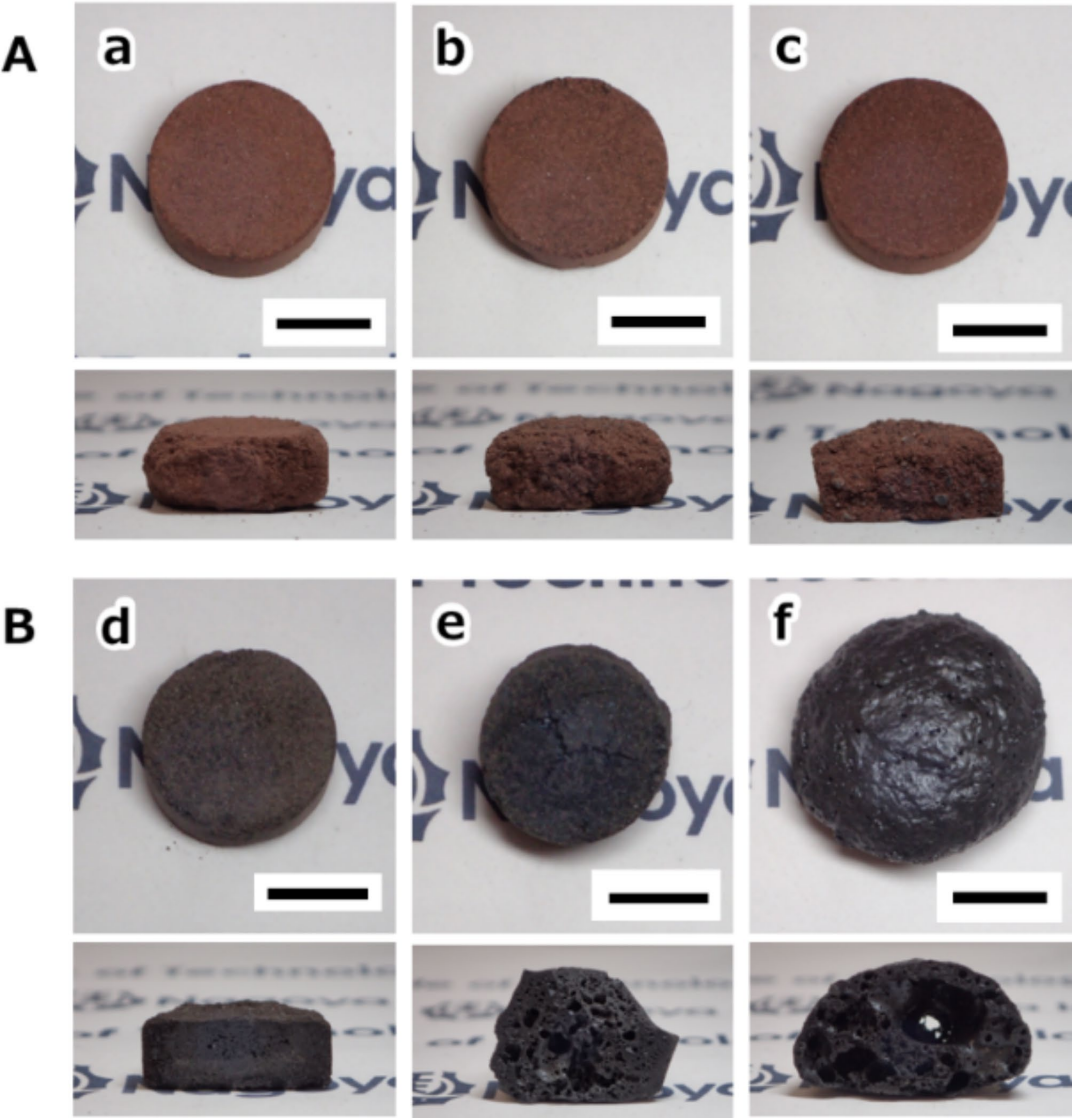
Photograph of obtained specimens via microwave heating in (**A**) atmospheric (air) and (**B**) vacuum conditions with varying treatment temperature: (a, d) 1000 ^o^C, (b, e) 1025 ^o^C, and (c, f) 1050 ^o^C. Scale bar indicates 10 mm.

Figure 2 presents photographs of specimens prepared by microwave heating in different atmospheres (air and vacuum). In the temperature range from 1000 to 1050 °C, the atmospheric heating resulted in the reddish-brown specimens with a too-brittle structure (not well sintered) (see Fig. 2A). Besides, the sintering started above 1050 ^o^C, and the molten structure was formed by the heating at over 1100 ^o^C (Fig. S2). In contrast, the resultant specimen prepared by microwave vacuum heating at 1000 °C was dense with less change in the shape from the compact (Fig. 2B). The coloration difference should be derived from the valence states of iron in the compounds (i.e., reddish-brown: hematite [Fe^3+^]_2_O_3_, black: magnetite [Fe^3+^]_2_[Fe^2+^]O_4_). However, increasing treatment temperatures drastically changed the appearance, even by just 50 °C. The heating at 1050 ºC caused the dome-like shape specimen to have many visible pores. The diameter change of about twice should cause a severe deterioration in mechanical strengths and make it hard to use as bricks for pavements or protective walls due to its less shape-retaining ability.

**Fig. 3:**
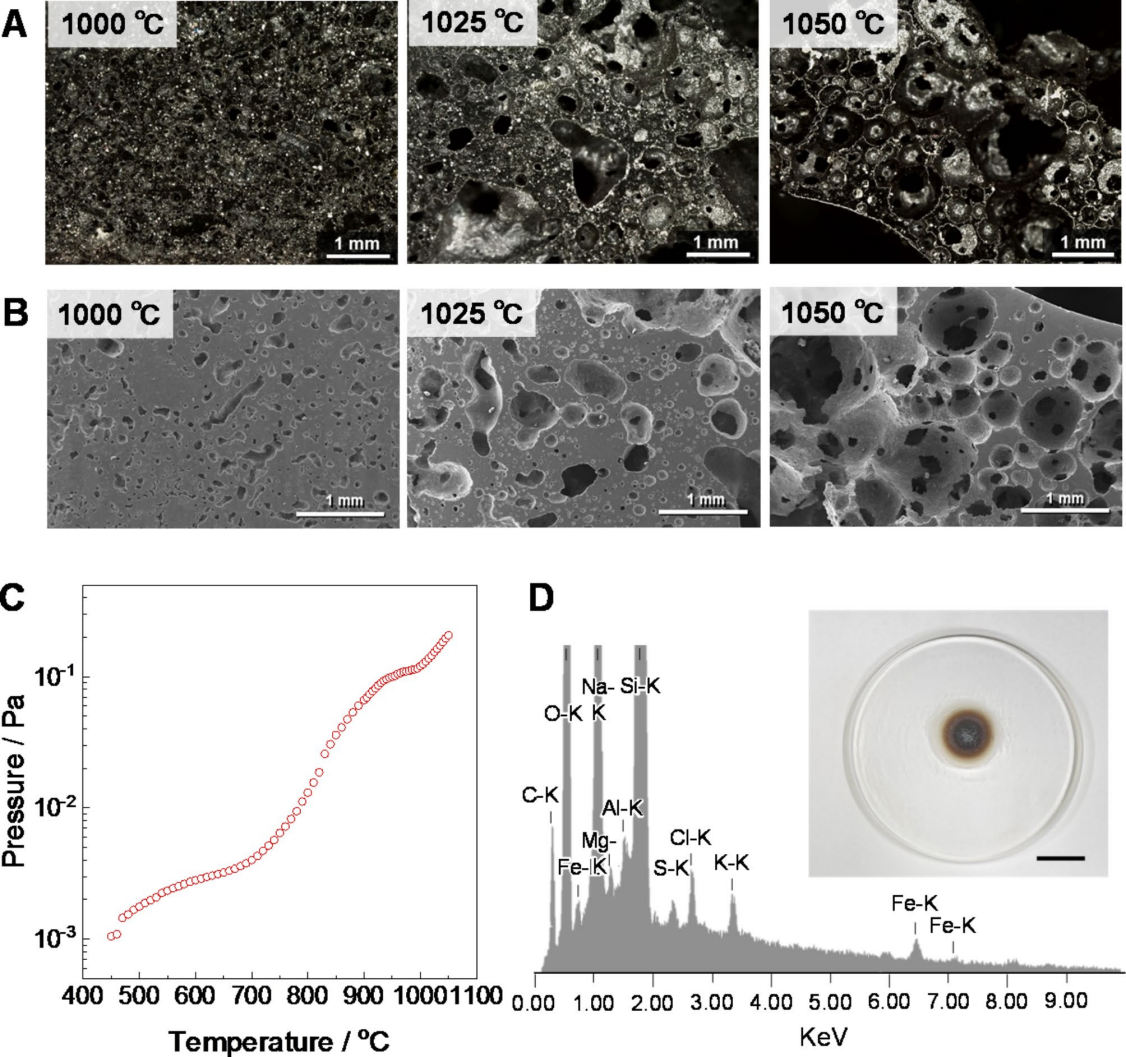
Microstructure of obtained specimens: (**A**) optical microscope and (**B**) SEM images of polished cross-section with a low magnification. (**C**) Vacuum pressure during microwave heating. (**D**) EDS analysis of deposited compounds inside a quartz glass container. Inset indicates photograph of deposited compounds.

As the microstructure of the specimens prepared by the vacuum heating, countless pores of several hundred µm size were microscopically found (see Fig. 3A, B). Monitoring the vacuum pressure during microwave heating clarified the stepwise degassing dynamics from the silicate compounds (Fig. 3C): The pressure gradually elevated at over 700 °C and sharply changed again above 1000 ^o^C. It consequently increased by two orders of magnitude from 500 to 1050 ^o^C. After the end of the experiment, the condensed volatile compounds with the dark brown color were deposited inside a quartz glass container (inset in Fig. 3D). The EDS analysis characterized that the compounds were mainly composed of Si and Na (73.53 and 21.01 mass%, respectively) (the details are summarized in Table S2). Besides, the volatile species, including Cl and S as minor elements, would cause an increase in vacuum pressure at 700–1000 ^o^C, whereas a sharp change in vacuum level occurred above 1000 ^o^C due to a thermal pyrolysis of silicates^16^. The volatilization caused an increase in the true density with elevated treatment temperature in the vacuum conditions (Fig. S3). In contrast, true density was not increased in the specimen prepared under the atmospheric conditions. Notably, Fe element of 2.00 mass% was detected, never reported in similar studies. This indicates that the self-heating of iron-containing compounds such as magnetite oxides preferentially involves thermal runaway owing to selective microwave absorption, leading to a drastic change compared with the atmospheric conditions, as described below.

### Microwave absorption capacity

**Fig. 4:**
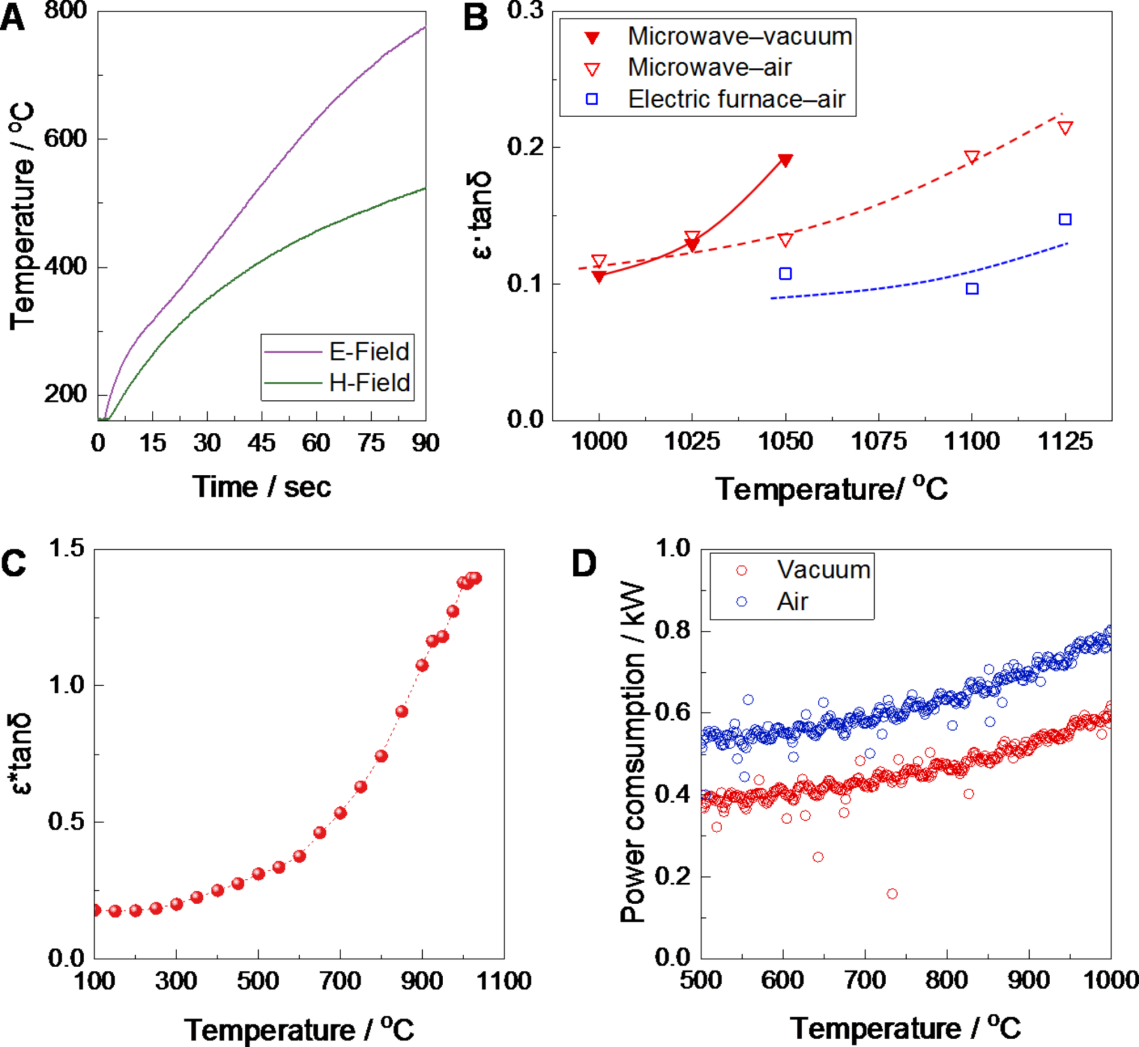
(**A**) Self-heating behavior applied in microwave electric/magnetic fields. Dielectric properties at 2.45 GHz: (**B**) measurement of obtained specimen at room temperature and (**C**) In-situ measurement of radical change during microwave vacuum heating, (**D**) Microwave power consumption monitored during heating.

Generally, eddy current loss, hysteresis loss, and dielectric loss are responsible for heat generation by the direct interaction of material–microwave electromagnetic fields^21^. A single-mode microwave applicator investigated the contribution of microwave electric/magnetic field to the heating (Fig. 4A). As a result, applying both electric and magnetic microwave fields considerably elevated the sample temperature. The magnetite phase performs superior permeability and permittivity than the hematite phase^22^ and has higher dielectric properties than other minerals^23^. Thus, the magnetite phase should be responsible for an excellent response to electric and magnetic fields, dominantly contributing to elevating microwave absorption capacity. In our case, the influence of hysteresis loss is negligible on rising temperature since the treatment temperature reached over 1000°C during the heating. The microwave absorption capacity was further evaluated by the dielectric properties of the specimen obtained through various conditions: treatment temperature (1000–1200°C), atmosphere (vacuum, air), and heating processes (microwave heating, conventional heating). Figure 4B represents the change in the product of relative permittivity (ε’) and dielectric loss angle (tanδ = ε”/ε’), plotted as a function of the treatment temperature. The values of ε*tanδ displayed the temperature dependence. Notably, the specimen obtained by microwave heating in the vacuum condition (e.g., MW-v-1050) exhibited superior absorption capacity, which was twice higher than conventional heating in the atmospheric condition (e.g., EF-a-1050). Therefore, the preferential formation of the magnetite phase in vacuum heating would dominantly cause high microwave absorption capacity. Moreover, the in-situ measurement of ε*tanδ proved that the dielectric constants gradually increase above 600–700 ^o^C, as shown in Fig. 4C. It is worth noting that the value at 1000 ^o^C increased one order of magnitude. Similarly for other regolith simulants^24^, the FJS-1 simulant exhibited that the value change in tan δ had quadratic temperature dependence as well as true density (**Fig. S4**). Additionally, the microwave dielectric constant of iron oxide (magnetite^25^, magnetite concentrate^26^, hematite^27^) exhibits strong temperature dependence. Specifically, it is noted that the increase begins above 500 ℃, with a significant rise occurring around 800℃. A similar behavior was observed in our work. Figure 4D compares the microwave output power monitored during the heating in the vacuum and atmospheric conditions. Microwave-vacuum heating achieved relatively lower power consumption. Besides, the output value decreased by 4.6% in the second run (**Fig. S5**). It suggests that the self-enhancement of the absorption capacity due to the chemical compositional change is a specific feature that cannot be realized in other processes. Evidentially, the evolved compounds from silicates presented the presence of Fe elements, indicating localized heating owing to the selective microwave absorption by iron-containing compounds such as magnetite. We calculated the skin depth (δ) and microwave penetration depth (D) of magnetite particles, which are associated with the microwave magnetic and electric fields, respectively (The detailed calculation procedure is included in SI. The parameters used in the calculations are listed in **Table S3**). The calculated results indicate that the skin depth of magnetite at ambient temperature is about δ = 74 μm, whereas the penetration depth is approximately D = 0.04 m. These are far larger than the magnetite crystals seen in Fig. 1. Some researchers have discussed the influence of the particle size to skin depth ratio (d/δ) on microwave heating efficiency^28^. Jie et al. reported that when d/δ>>1, microwaves are reflected off the particle’s surface, resulting in a substantial reduction in heating efficiency. In contrast, when d/δ<<1, although heat can fully penetrate the particle inside, the degree of heat generation is minimal. They concluded that the condition of d/δ = 1 achieves high heating efficiency^29^. Amini et al. examined the correlation between d/δ of Fe_3_O_4_ and the efficiency of microwave heating. They indicate that in a reduction process of Fe_3_O_4_ in an H_2_ atmosphere, the reaction efficiency diminishes when d/δ is either very small or excessively large^30^. It has been found that at high temperatures, particularly over 500 ℃, the dielectric constant and electrical conductivity significantly rise, simultaneously with a decrease in skin depth^26,27^. Thus, changes in particle size resulting from the crystal growth of the magnetite phase in elevated temperature regions would substantially influence microwave heating behavior. In addition, a broad particle size distribution would lead to non-uniform heating, potentially resulting in defect formation owing to thermal runaway. It should be noted that the microwave heating process would offer further benefits because of nano-phase Fe in lunar regolith^10,31^, which would considerably contribute to highly efficient self-heating by eddy-current loss or hysteresis loss below 1000 ^o^C.

### New thermal design of microwave heating for enhancing mechanical properties

**Fig. 5:**
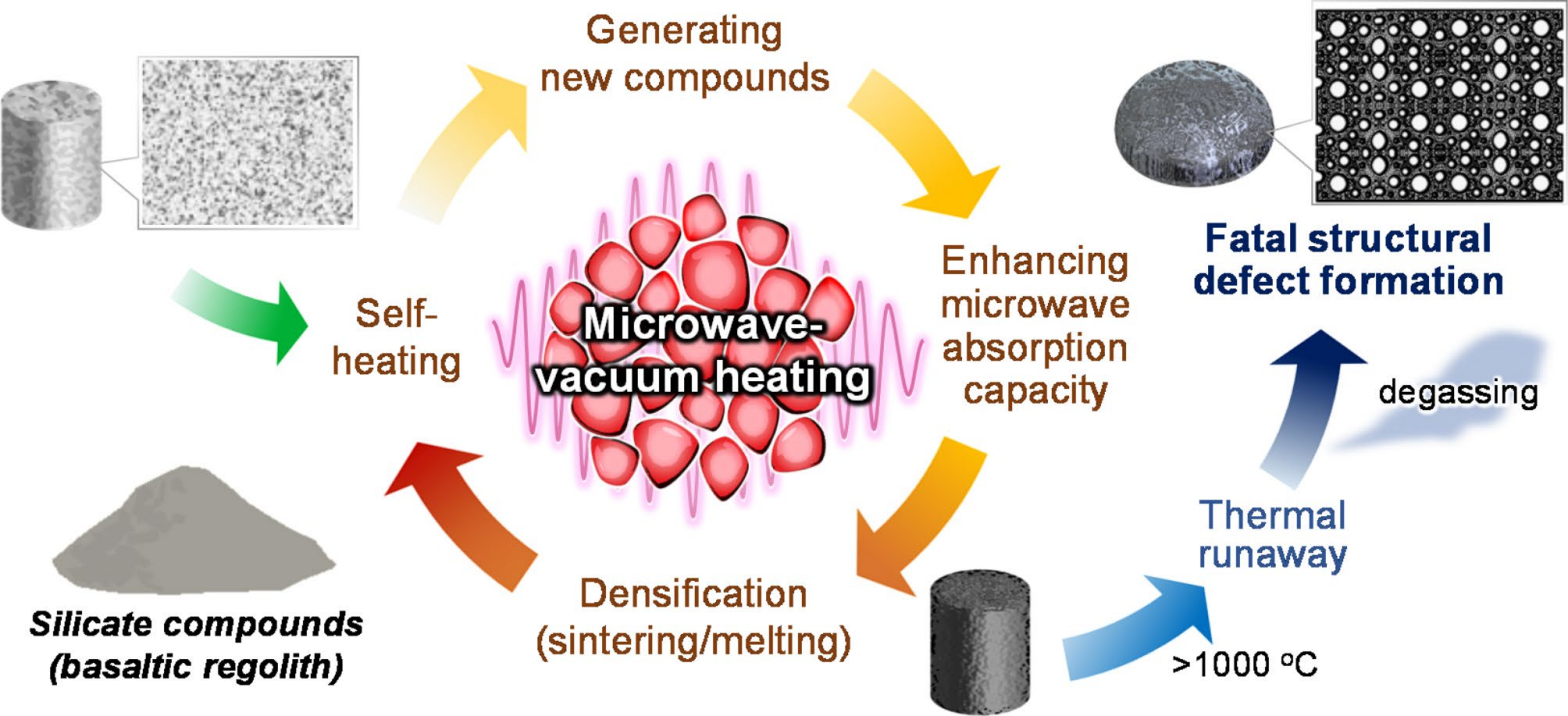
Possible reaction phenomena during microwave vacuum heating for basaltic regolith,

Based on the results, we presume the possible phenomena assisted by microwave vacuum heating for basaltic regolith, as follows (Fig. 5): Firstly, the temperature of silicates gradually increases by a combination of self-heating of both FJS-1 and a SiC susceptor at a low-temperature region (< 600 ^o^C), followed by preferentially forming reduced iron oxides (i.e., magnetite phase) as new compounds [Phase 1]. It allows more highly efficient self-heating owing to enhancing microwave absorption capacity, compared to the synthesis under atmospheric conditions. Then, efficient mass transport and atomic diffusion in molten facilitate the densification [Phase 2]. This is a unique process beyond other methods; however, thermal runaway^32^ caused degassing with volatile species (e.g., oxides of Si, Na, and Fe) due to thermal pyrolysis of silicates, consequently generating numerous pores as catastrophic structural defects [Phase 3]. Thus, enhancing microwave absorption capacity offers both benefits and negative impacts. Holding at high temperatures (> 1000 ^o^C) helps to densify the bulk by forming a three-dimensional network structure of molten. In contrast, catastrophic defects inevitably form by degassing owing to thermal pyrolysis, causing a considerable deterioration in mechanical strength. The characteristic behavior in vacuum heating has rendered microwave technologies infeasible for fabricating building materials for lunar base construction.

**Fig. 6:**
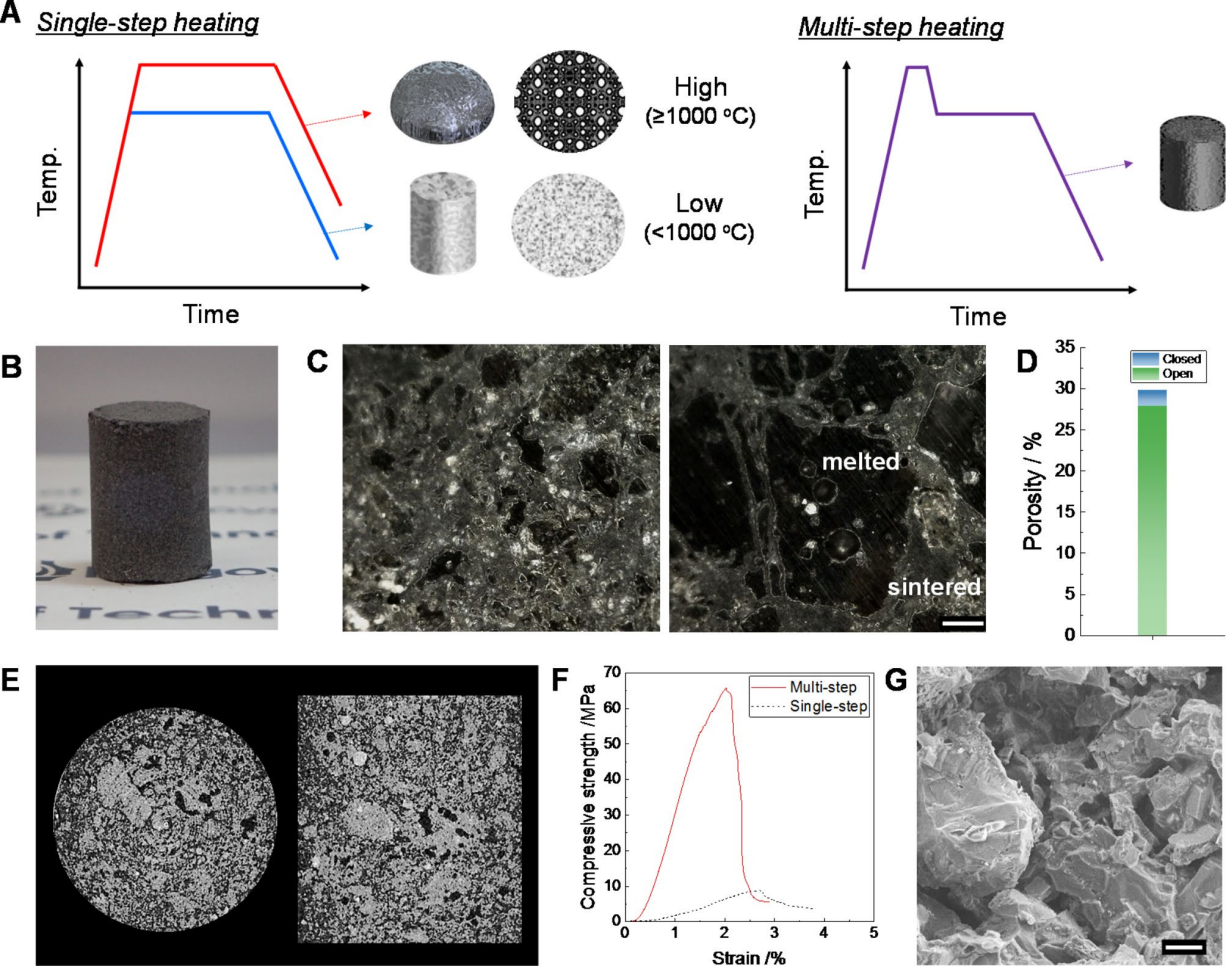
(**A**) Typical temperature program for single-step and multi-step heating. (**B**) Photograph of specimen obtained through multi-step heating. (**C**) Optical microscopy images of polished cross-section (scale bar is 100 μm). (**D**) Porosity. (**E**) X-ray CT images. (**F**) Compressive strength test. (Reference specimen fabricated by single-step heating: MW-v-1000). (G) SEM image of fracture surface.

Here, we propose a new thermal design concept to overcome the practical limitations of microwave technology in a vacuum atmosphere. For simultaneously achieving highly robust microstructure and suppression of pore formation, the multi-step temperature profiles were programmed, as typically illustrated in Fig. 6A. Especially for basaltic-regolith, the first step should be conducted at a higher temperature where melting starts (i.e., > 1000 ^o^C) with a short holding-time (≤ 5 min), whereas the holding temperature should be much lower than the first step at the second-steps. Optimizing the temperature profile successfully enables it to keep the compact shape without severe deformation despite undergoing high-temperature treatment over 1000 ^o^C (the photograph is shown in Fig. 6B). The obtained bulk specimen consisted of a hybrid microstructure with the molten and sintered phase with the relative density of approximately 70%, in which the open and closed porosity were 27.8 and 1.9%, respectively (Fig. 6C and D). X-ray CT image confirmed the partial densification in the specimen (Fig. 6E). In addition, the thermal pyrolysis of silicate compounds resulted in the formation of closed pores, which were mostly found in the molten part. Figure S6 compares the specimens obtained through single-step heating (MW-v-1000) and multi-step heating. The peak below 10 μm range is attributed to the interparticle gaps, while degassing owing to thermal pyrolysis of silicate compounds caused the formation of the larger pores (above 70 μm). The multi-step heating program facilitated the formation of the molten glass components with the three-dimensional network in the first-step at temperatures exceeding 1000 ℃, followed by a quick temperature reduction of over 50 ℃ in the second step to mitigate thermal pyrolysis and to prevent the emergence of critical defects caused by thermal runaway. Consequently, the specific microstructure considerably enhanced the mechanical strength of the resultant block without the undesirable changes in shape. Figure 6F plots the stress-strain curves in the compressive strength test. Despite the low relative density, the specimen obtained via multi-step heat treatment demonstrated remarkable enhancement in the compressive strength up to 65 MPa, compared with the single-step heat treatment (~ 35 MPa). Besides, Young’s modulus increased by an order of magnitude from 0.36 GPa (single-step) to 5.81 GPa (multi-step). Besides, cleavage fracture surfaces were observed as a characteristic feature of molten glass (Fig. 6G). Thus, the primary factor contributing to the bulk strength would be the molten glass components with the three-dimensional network. Table 1 compares performance with the reported studies^20,33–36^. The microwave-fabricated specimens exhibited outstanding performance even under high vacuum heating without vigorous hydrostatic cold press as a pre-treatment.

**Table 1:** Comparison of performance with similar reported studies.

Regolith simulant	Compressive strength (MPa)	Method	Atmosphere	Temperature (^o^C)	Pre-treatment	
FJS-1	~ 65	microwave	vacuum	~ 1025	axial pressing (< 30 MPa)	**This work**
FJS-1	47	microwave	air	1125	n.a.	Ref^20^
JSC-1 A	250	electric furnace	air	1120	axial pressing (40 MPa)	Ref^26^
JSC-1 A	150	electric furnace	vacuum	1100	hydrostatic pressing (255 MPa)	Ref^27^
HUST-1	~ 68	electric furnace	vacuum	1050	hydrostatic pressing (255 MPa)	Ref^28^
FJS-1	~ 38	electric furnace	vacuum	~ 1175	axial pressing (40 MPa)	Ref^29^

We highlight the practical challenges and some solutions to ensure feasibility and compatibility in the large-scale implementation for fabrication of construction materials using microwave technology.

#### Further validation of correlation between mineral composition and microwave heating behavior

This study revealed that the microwave heating offers distinct advantages over alternative technologies, including enhanced microwave absorption and heating efficiency attributed to structural changes in the compound, especially iron oxides. However, overheating induced by the runaway phenomenon accelerates the thermal pyrolysis of the silicate compound, leading to gas generation and the formation of numerous defects (pores), which constitute significant flaws in the bulk of the materials. Considering the aforementioned, the locations of regolith mining sites and the chosen regolith simulant may exhibit markedly different mineral compositions, leading to considerable variations in microwave heating behavior. Lunar areas are broadly divided into two categories: mare and highland. The lunar mare is basaltic and consists of five major minerals: plagioclase (anorthite), pyroxene (orthopyroxene [(Mg, Fe)SiO_3_], clinopyroxene [Ca(Fe)SiMg)Si_2_O_6_]), olivine [(Mg, Fe)_2_SiO_4_], and titanite (ilmenite [FeTiO_3_]). In contrast, the highland comprises anorthositic rocks, with other minerals such as pyroxene and olivine (a few volume percent)^5^. Additional work is required particularly using Highland regolith simulants with chemical (mineral) compositions distinct from Mare regolith simulants (FJS-1). The impact of these minerals on microwave heating behavior will be addressed in a forthcoming publication.

#### Bridging gaps between simulants and actual regolith

Regolith has distinctive features compared to the developed simulants by combining terrestrial minerals, resulting from its specific formation mechanism^6^. Although many regolith simulants have been developed that mimic the chemical composition, particle size, size distribution, and various mechanical properties of lunar regolith, they are deficient in compounds unique to regolith (e.g., np-Fe^10,31^, agglutinates^37^) due to the difficulties to imitate the lunar environment (i.e., vacuum, low gravity, extreme temperatures, and significant exposure to solar wind and micro-meteorite impacts). Taylor et al. have addressed that the existence of nano-scale metallic iron spheres (typically, < 10 nm) in agglutinitic glass would significantly influence the characteristic of microwave absorption^10^. Thus, the development of extremely high-fidelity simulants is high-priority challenging. Moreover, process informatics (PI) incorporated with artificial intelligence (AI) can elucidate the principal parameters in the complex correlation between material compositions and microwave absorption behavior, and produce superior building materials by deducing the most optimal heating program according to the composition. This would significantly accelerate the development of residential facilities and paved roadways, leading directly to the success of hummanned exploration and extended stays on the Moon.

#### Durability test under severe conditions and establishment of continuous manufacturing/automation technology

In the lunar environment, cosmic rays, meteorite impacts^38^, and thermal-induced stress resulting from temperature variations^39^ are the primary processes that lead to weathering and breakdown. It has been reported that thermal fatigue driven by accumulated strain may result in failure when regolith rocks undergo elastic deformation due to temperature variations between day and night^40^. Therefore, evaluating the thermal shock resistance of materials under extreme temperatures is crucial. Furthermore, it is required for a more thorough examination using the microgravity test provided by a series of parabolic flights or the International Space Station in order to verify the impact of low gravity on microwave heating behavior and degassing dynamics associated with microstructure formation and mechanical properties. Moreover, given the severe environment on the Moon, it is imperative to advance technologies for resource surveying (composition detection and screening), continuous manufacturing, and the automation of construction systems for quickly and precisely assembling buildings to enhance crew safety and expedite construction. Integrating remote-controlled and automated robotic systems^41,42^ will be helpful in solving these challenges.

## Conclusion

In this study, we comprehensively investigated the microwave heating behavior of basaltic regolith simulant (FJS-1) in a high-vacuum atmosphere to enhance the feasibility of microwave technologies in building material manufacturing for lunar base construction. Specifically, we focused on the changes in chemical structure, microstructure, and microwave absorption properties. The XRD, SEM-BSE, and Raman microscopic analysis proved that the vacuum heating associated the compositional changes to intermediate sodian anorthite and facilitated the crystallization of magnetite (Fe_3_O_4_) rather than the hematite (Fe_2_O_3_) phase formation. Besides, the apparent shape of the resultant specimen presented a radical change by microwave vacuum heating in the 1000–1050 °C range. It caused catastrophic failure in the microstructure by forming countless pores of several hundred µm size. The degassing dynamics were revealed by monitoring the vacuum pressure during microwave heating: the volatile compounds, including Cl and S, would cause an increase in vacuum pressure at 700–1000 ^o^C, whereas a sharp change was observed above 1000 ^o^C due to thermal pyrolysis of silicates (mainly composed of Si and Na). Moreover, the contribution of the microwave electric/magnetic field to heat generation was investigated by a single-mode microwave applicator: applying both electric and magnetic microwave fields contributed to elevating the sample temperature considerably. Notably, the microwave-vacuum synthesized specimen exhibited superior microwave absorption capacity, where the values of ε*tanδ were twice higher than conventional heating in the atmospheric condition since the preferential formation of the magnetite phase in vacuum heating would dominantly cause high microwave absorption capacity. In addition, we first reported the in-situ measurement for dielectric properties of regolith simulant during microwave heating under a vacuum condition: the ε*tanδ increased one order of magnitude from R.T. to 1000 ^o^C. Furthermore, microwave-vacuum heating achieved lower power consumption, where the output value decreased by 4.6% in the second run. Evidentially, the evolved compounds from silicates presented the presence of Fe elements, indicating localized heating owing to the selective microwave absorption by iron-containing compounds such as magnetite. In the last section, we propose a new thermal design concept to overcome the practical limitations of microwave technology in a vacuum atmosphere. The multi-step temperature profiles successfully enable it to keep the compact shape without severe deformation despite undergoing high-temperature treatment over 1000 ^o^C. Despite the low relative density (approximately 70%), the specimen obtained via multi-step heat treatment demonstrated outstanding performance even under high vacuum heating: the compressive strength was up to 65 MPa, whereas ~ 35 MPa by the single-step heat treatment. Besides, Young’s modulus increased by an order of magnitude from 0.36 GPa (single-step) to 5.81 GPa (multi-step). They were comparable to world-class mechanical properties reported in similar studies using a vigorous hydrostatic cold press as a pre-treatment.

## Experimental section

### Microwave vacuum heating

Fuji Japanese Simulant (FJS-1; Lot. 1901, Shimizu corp.)^43^ was used as a raw material. The chemical composition of FJS-1 is shown in Table S4. A 2.45 GHz multi-mode industrial microwave furnace (magnetron, maximum 3.0 kW, MWK-B-3.0, Takasago) was used for microwave heating. Connecting a custom-made quartz container (diameter 100 mm x height 125 mm) to a turbo-molecular pump unit (ST220FV, Osaka Vacuum, Ltd.) enables the execution of heating experiments under high vacuum conditions. A 5 g compact of FJS-1 was first formed using a uniaxial press (pressure ≤ 30 MPa). Subsequently, the compact was placed in a handmade insulated kiln, located into the quartz container, and sealed with a round quartz plate. The inside wall of the kiln was coated with silicon carbide. The apparatus is shown in Fig. S7. The vacuum was monitored using an ionization vacuum gauge, and upon achieving a high vacuum (10^− 3^ Pa), the temperature programming began. During microwave heating, the sample surface temperature was measured using a radiation thermometer via an aperture in the kiln’s top. The temperature was regulated between 1000 and 1050 ℃. A typical temperature program and the recorded temperatures are shown in Fig. S8. The heating rate was 20 °C/min, and the duration of holding was 15 min for all heating settings. The reference samples were prepared at atmospheric pressure, maintaining the same thermal history.

### Characterization

The crystal structure was identified by an X-ray diffractometer (XRD; Ultima-IV, Rigaku). The chemical structure was evaluated using Raman microscopy (inVia Inspect, Renishaw). The cross-section of the microstructure in the prepared specimens was observed by optical microscope (DVM6, Leica Microsystems Ltd.) and scanning electron microscope (SEM; JCM-6000Plus, JEOL). True density measurement was conducted by using a Pycnometer (AccuPyc II, Micromeritics Instrument). The dielectric properties were measured by using a 2.45 GHz microwave cavity resonator (AET, Inc.), equipped with a vector Network Analyzer (VNA; MS46122B, Anritsu). The detailed experimental procedure and calculation method are included in SI. The internal microstructure of the prepared specimen was observed by an X-ray computed tomography (X-ray CT: inspeXio SMX-100CT, Shimadzu). The relative density and porosity were evaluated using Archimedes’ method. The pore size distribution was measured by the mercury porosimeter (AutoPore V, Micromeritics Instruments Corp.). The mechanical properties of the obtained specimens were evaluated using an autograph (AGX-V, Shimadzu) equipped with 50 kN load cells. The test speed was 0.5 mm/min. Young’s modulus was calculated from the stress-strain curve.

## Electronic supplementary material

Below is the link to the electronic supplementary material.

Supplementary Material 1

## Data Availability

The data that support the findings of this study are available from the corresponding author, Assoc. Prof. Dr. Takashi Shirai, upon reasonable request.
