# Supplementary material for: Multi-step thermal design of microwave vacuum heating to basaltic regolith simulant towards lunar base construction
Source: Sci Rep. 2024 Nov 15;14:28231. doi: 10.1038/s41598-024-79504-x (PMC11568225; doi:10.1038/s41598-024-79504-x)
Supplement: Supplementary file 1 — Supplementary Material 1 [file 41598_2024_79504_MOESM1_ESM.docx]

**Supplementary Information**

**Multi-step thermal design of microwave vacuum heating to basaltic regolith simulant towards lunar base construction**

Kunihiko Kato ^a^ and Takashi Shirai ^a^*

^a^ Advanced Ceramics Research Center, Nagoya Institute of Technology, Gokiso, Showa-ku, Nagoya, Aichi 466-8555 Japan

*^*^Corresponding author e-mail:*  [shirai@nitech.ac.jp](mailto:shirai@nitech.ac.jp)

**Experimental Section**

Characterization of dielectric properties

Dielectric properties were evaluated using a cylindrical cavity resonator. Powder samples were placed into quartz tubes and inserted into the resonator for this measurement. The complex permittivity was assessed and computed using specialized measurement and analysis tools, derived from the variations in resonant frequency and Q-value of the resonator (Perturbation theory). The following equation (eq. S1) expresses the change in resonance frequency and Q-value as a result of sample insertion.

| $\left( \frac{f_{2}-f_{1}}{f_{1}} \right)+j\left( \frac{1}{2Q_{2}}-\frac{1}{2Q_{1}} \right)=-\left( \frac{{\varepsilon'}_{r}-1}{2} \right)\frac{\int_{V_{s}} E_{2}\cdot E_{1}dV}{\int_{V_{c}} \left\vert E_{1} \right\vert^{2}dV}$ | (S1) |
| --- | --- |

where f_1_ and f_2_ denote the resonance frequencies, while Q_1_ and Q_2_ represent the Q values before and after the sample insertion, respectively. ε_r_ indicates the complex permittivity of the sample, E_1_ and E_2_ mean the electric field vectors inside the resonator before and subsequent to sample insertion.

Eq. S1 can be separated into real and imaginary components as shown in Eq. S2 to S4.

| $\left( \frac{f_{2}-f_{1}}{f_{1}} \right)=-\left( \frac{{\varepsilon'}_{r}-1}{2} \right)C$ | (S2) |
| --- | --- |
| $\frac{1}{Q_{2}}-\frac{1}{Q_{1}}={\varepsilon"}_{r}C$ | (S3) |
| $C=\frac{\int_{V_{s}} E_{2}\cdot E_{1}dV}{\int_{V_{c}} \left\vert E_{1} \right\vert^{2}dV}$ | (S4) |

where V_c_ and V_s_ correspond to the total volume of the resonator and the volume within the sample, respectively. Perturbation theory considers the parameter C in Eq. S4 as invariant to the dielectric constant of sample. The parameter C is computed using an electromagnetic field analysis program (SIMULIA CST Studio Suite), provided by AET, Inc.. A cylindrical cavity resonator with TE_111_ mode was used for in-situ measurements of dielectric constant during microwave heating. The VNA was operated under S11 mode to assess resonance frequency and Q-value. Since the tested powder samples possess different filling ratios, this influences the relative permittivity and dielectric loss angle, as the reviewer pointed out. Thus, the following approach is used to consider the effect of a packing density of powder to eliminate the impact of air on the underestimation of the measurement values. Given that the electric field surrounding the glass capillary is uniform, the dielectric constant of the analyzed sample could be determined from the volume ratio of the sample and the void inside the glass capillary. Here ε_a_ and A are defined as the apparent value of dielectric constant and the volume ratio. The effective dielectric constant of the measured sample, ε_s_, are expressed by eq. S5.

| $\varepsilon_{a}-1=(\varepsilon_{s}-1)\cdot A$ | (S5) |
| --- | --- |

In addition, eq. S5 can be transformed into eq. S6.

| $\varepsilon_{s}=\frac{\varepsilon_{a}-(1-A)}{A}$ | (S6) |
| --- | --- |

Volume ratio A is derived from the true density (measured by Pycnometer) and the apparent density of the powder, which are determined using the filling weight and the volume inside the tube.

Calculation of skin depth and penetration depth of magnetite particles

The skin depth (δ) and microwave penetration depth (D) of magnetite particles associated to the microwave magnetic and electric fields are expressed by equations S7 [1] and S8 [2], respectively.

| $\delta=\sqrt{\frac{1}{\pi\mu\sigma f}}$ | (S7) |
| --- | --- |
| $D= \frac{c}{2\sqrt{2}\pi f}\left\{ {\varepsilon"}_{r}{\mu"}_{r}-{\varepsilon^{'}}_{r}{\mu^{'}}_{r}+\left[ \left( {\varepsilon^{'}}_{r} {\mu^{'}}_{r} \right)^{2}+\left( {\varepsilon"}_{r} {\mu"}_{r} \right)^{2}+\left( {\varepsilon^{'}}_{r}{\mu"}_{r} \right)^{2}+\left( {\varepsilon"}_{r}{\mu^{'}}_{r} \right)^{2} \right] \right\}$ | (S8) |

Where σ is electrical conductivity (S/m). ε’_r_ and ε”_r_ means real part and imaginary part of complex relative permittivity, whereas μ’_r_ and μ”_r_ represents real part and imaginary part of complex relative permeability, respectively. c and f indicate speed of light (=2.99792×10^8^ m/s) and microwave frequency (Hz), respectively..

**Supporting Figures**


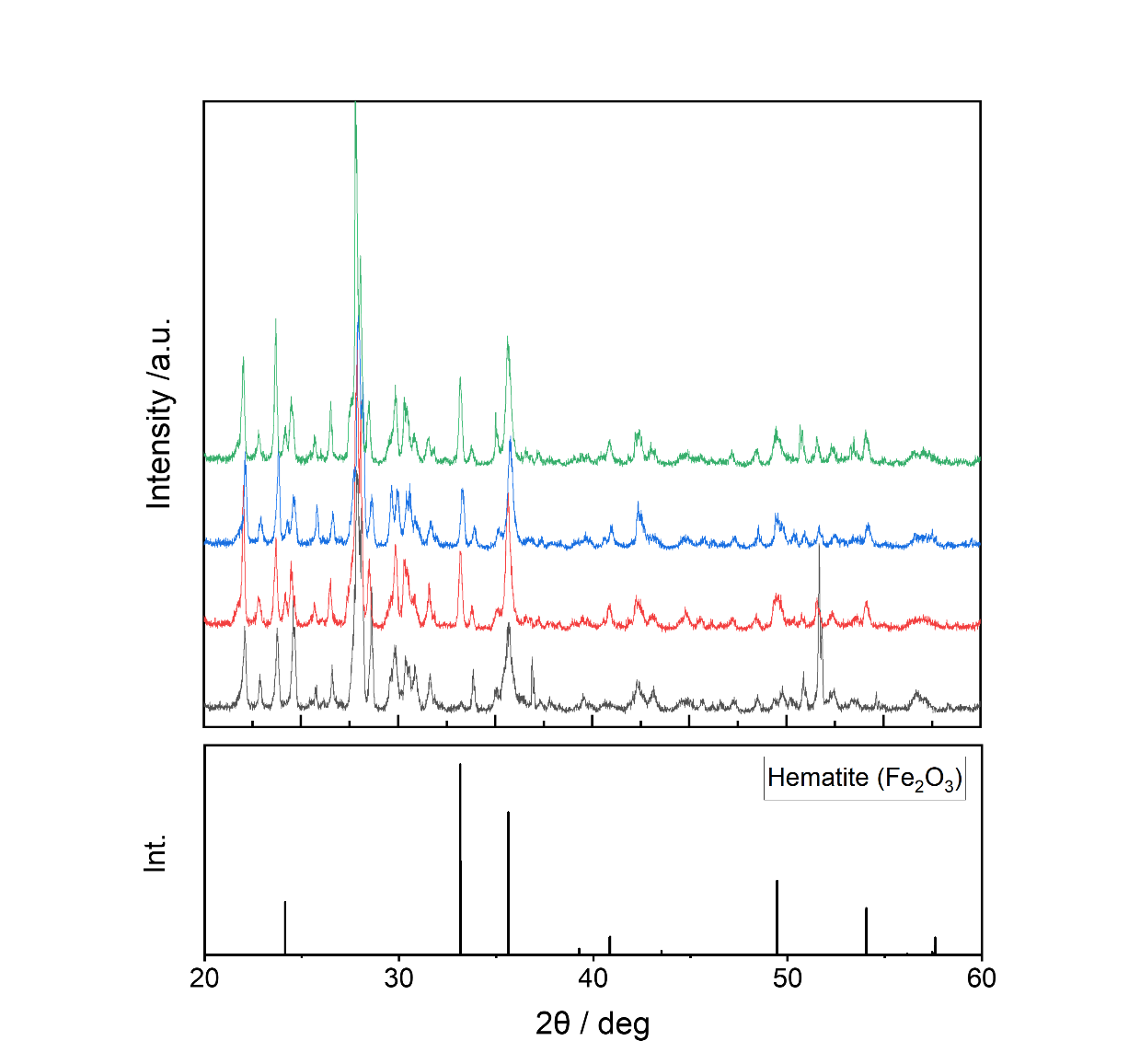


**Figure S1.** PXRD patterns of obtained specimens via microwave heating in atmospheric conditions (air).

**Figure S2.** Photograph of obtained specimens via microwave heating in atmospheric conditions (air) up to at 1150 ^o^C.

**Figure S3.** Comparison of true density of specimen: vacuum vs. air.

**Figure S4.** Relationship between true density and dielectric loss angle,

**Figure S5.** Power consumption change by repeating microwave heating.

**Figure S6.** Pore size distribution: single-step *vs.* multi-step heating program.


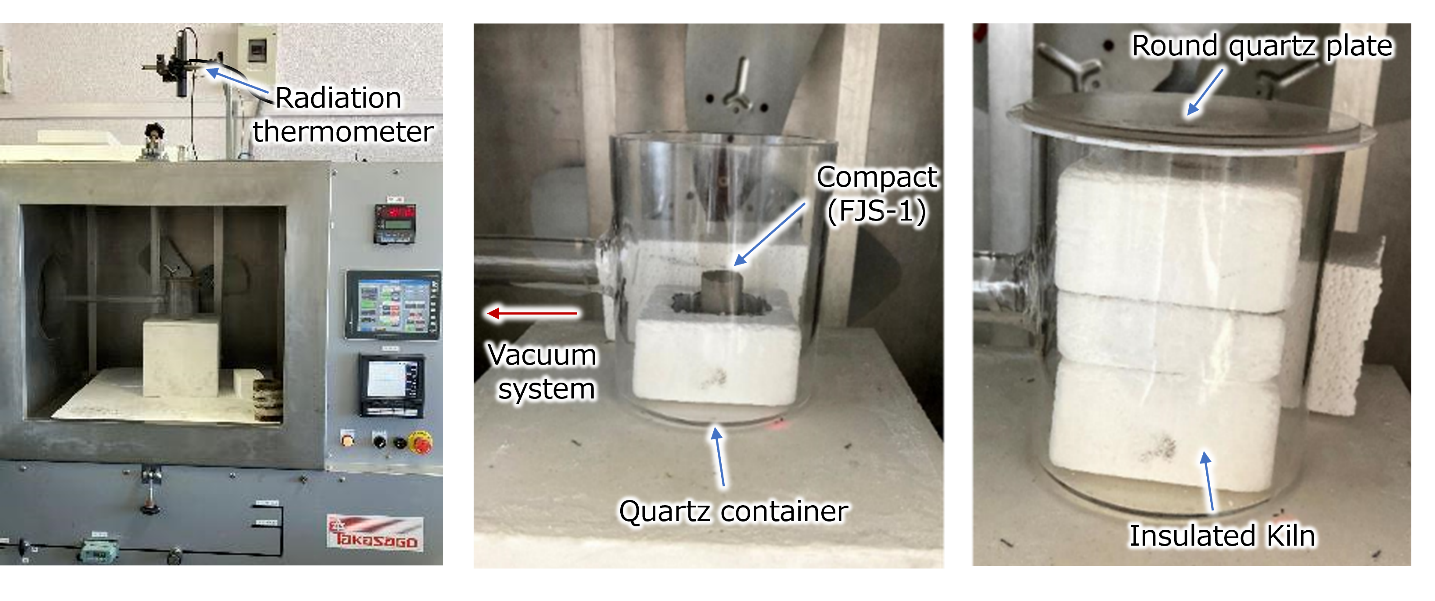


**Figure 7.** Experimental setup for microwave vacuum heating.

**Figure 8.** Typical temperature program and recorded temperature (single-stage heating).

**Supporting Tables**

**Table S1.** Classification of methods for building materials fabrication using regolith.

| Method | Technology | Raw materials | Features |
| --- | --- | --- | --- |
| Solidification “Chemical reaction” | - Concrete - Geopolymer | - **Regolith (aggregates)** - Water glass - Alkaline solution - Organic compounds  (e.g., surfactant） | [Advantages]   - - Abundant experience and know-how   - Capability of production in mild environments   [Disadvantages]   - Require scarce resources such as water and organic materials - Require facilities to prevent water evaporation before curing - Need to supply missing Al components from other materials - Relatively low strength compared to other technologies |
| Compressing | - Cold pressing | - **Regolith** - Clay mineral | [Advantages]   - Operate just pressing   [Disadvantages]   - Use clay minerals (Suitable for use on Mars) |
| Sintering/Melting | - Electric furnace heating - Laser heating (additive manufacturing) - **Microwave heating** | - **Regolith** | [Advantages]   - Use only regolith as raw material - High strength   [Disadvantages]   - Require know-how for temperature control in high-temperature range |

**Table S2.** Chemical composition of volatile gas evaluated by EDS.

|  | mass% |
| --- | --- |
| Si | 73.53 |
| Na | 21.01 |
| Fe | 2.00 |
| K | 1.25 |
| Cl | 1.29 |
| S | 0.47 |
| Mg | 0.38 |
| Al | 0.18 |

**Table S3.** Summary of parameters in calculations

| Parameter | σ  [Ref. 3] | ε’_r_  [Ref. 2] | ε”_r_  [Ref. 2] | μ’_r_  [Ref. 2] | μ”_r_  [Ref. 2] |
| --- | --- | --- | --- | --- | --- |
|  | 10000 | 14 | 1.5 | 1.9 | 1.5 |

**Table S4.** Chemical composition of FJS and regolith.

|  | A11 | A14 | A16 | Basalt | **FJS-1** |
| --- | --- | --- | --- | --- | --- |
| SiO₂ | 42.2 | 48.1 | 45.0 | 45.8 | 49.8 |
| Al₂O₃ | 13.6 | 17.4 | 27.3 | 14.6 | 19.9 |
| TiO₂ | 7.8 | 1.7 | 0.5 | 2.6 | 2.0 |
| FeO | 15.3 | 10.4 | 5.1 | 8.7 | 8.2 |
| Fe₂O₃ | － | － | － | 3.2 | 10.2 |
| MgO | 7.8 | 9.4 | 5.7 | 9.4 | 3.9 |
| CaO | 11.9 | 10.7 | 15.7 | 10.7 | 1.4 |
| Na₂O | 0.5 | 0.7 | 0.5 | 2.6 | 2.9 |
| K₂O | 0.2 | 0.6 | 0.2 | 1.0 | 0.7 |

**References**

1. Hossain, F., Turner, J. V., Wilson, R., Chen, L., de Looze, G., Kingman, S. W., Dodds, C. & Dimitrakis, G. State-of-the-art in microwave processing of metals, metal powders and alloys. *Renew. Sustain. Energy Rev.* **202**, 114650 (2024).
2. Hotta, M., Hayashi, M., Nishikata, A. & Nagata, K. Complex Permittivity and Permeability of SiO_2_ and Fe_3_O_4_ Powders in Microwave Frequency Range between 0.2 and 13.5 GHz. ISIJ Int. 49, 1443–1448 (2009).
3. Yoshikawa, N., Xie, G., Cao, Z. & Louzguine, D. V. Microstructure of selectively heated (hot spot) region in Fe_3_O_4_ powder compacts by microwave irradiation. *J. Eur. Ceram. Soc.* **32**, 419–424 (2012).
